# Influence of facial pattern on the aesthetic assessment of the inclination of the occlusal plane in the frontal view

**DOI:** 10.1007/s00784-025-06254-z

**Published:** 2025-03-11

**Authors:** Juan Cárdenas-Soria, Luis-Alberto Bravo-González, Ascensión Vicente

**Affiliations:** Unit of Orthodontics, University Dental Clinic, University of Murcia. C/ Marqués de los Vélez. Hospital Morales Meseguer, 2º Planta, 30008 Murcia, Spain

**Keywords:** Occlusal plane, Facial biotype, Aesthetic, Facial pattern, Canting

## Abstract

**Objectives:**

The aim of this study was to analyse the aesthetic perception by orthodontists, dentists and laypeople of the different inclinations on the occlusal plane in the frontal view in relation to facial biotype.

**Materials and methods:**

The three groups of assessors evaluated the aesthetics of images with occlusal plane inclinations of 0º, 2º and 4º in a mesofacial, dolichofacial and brachyfacial face. Scores were assigned from 1 to 10 (“1”: poor aesthetics and “10”: very satisfactory aesthetics). The data were evaluated by the Kruskal–Wallis test (p < 0.05) and the Mann–Whitney test (Bonferroni correction p < 0.016).

**Results:**

In meso- and dolichofacial faces, orthodontists considered 0º occlusal plane inclination to be significantly more aesthetic than 2º (mesofacial p = 0.004, dolichofacial p < 0.001) and 4º (p < 0.001), and 2º inclination more than 4º (p < 0.001). Dentists and laypeople rated 0º and 2º inclinations as significantly more aesthetic than 4º (p < 0.001) with no significant differences between 0º and 2º inclinations. For the brachyfacial face, the three groups rated 0º and 2º occlusal plane inclination as significantly more aesthetic than 4º inclination (p < 0.001), with 0º and 2º inclinations scoring similarly (p > 0.016).

**Conclusions:**

Orthodontists were more critical than dentists and laypeople when assessing mesofacial and dolichofacial facial types, whereas no differences were observed between the three groups for brachyfacial faces.

**Clinical relevance:**

The facial pattern influences the perception of the occlusal plane. Understanding these differences is crucial for clinicians, as it can enhance diagnostic accuracy and optimize treatment planning, leading to more personalized and effective orthodontic care.

## Introduction

Facial aesthetics are of great importance today. Attractive people are perceived as intelligent, self-confident and are more socially accepted [1, 2].

However, the relationship between facial attractiveness and facial components is not entirely clear. Sano and Kawabata found that eyes are essential for facial attractiveness [3]. However, Patusco et al. found that in facial attractiveness the most important role is played by the mouth and in second place, the eyes, followed by the chin and then the nose [4]. In any case, the assessment of facial beauty is subjective; therefore, in an attempt to guide clinicians in determining what is considered a beautiful face, a set of standards have been suggested for the assessment of facial proportions [5].

Furthermore, when assessing the aesthetics of a smile, the patient's vertical pattern should be considered, as the aesthetic impact of the same smile may be different on a short or long face [6]. Brachyfacial people are characterised by a broad face and strong chin, flat lip posture, low mandibular plane angle, and a straight profile. Dolichofacial people have long narrow faces with a high mandibular angle, convex profile, poor chin development, and an anterior–posterior face height imbalance. In contrast, mesofacial individuals are characterised by well-balanced facial features [7].

What’s more, different studies have observed that facial pattern influence the aesthetic perception of different smile features, such as midline deviation [8], the degree of gingival exposure [9], the width of the buccal corridors [7, 10, 11] or the labiolingual inclination and anteroposterior position of the incisors [12].

Motamedian et al. found that one of the features that has the greatest impact on the aesthetic perception of a smile is the inclination of the occlusal plane in the frontal view [13]. Some papers that study this feature base their assessments on isolated photographs of smiles [14, 15] and others on images of full faces [16–18]. On the other hand, there is controversy over whether the evaluator's profession affects the perception of the occlusal plane; some studies have found that it does influence perception [15, 18], while others disagree [16].

To our knowledge, the influence of facial pattern on the aesthetic perception of different degrees of occlusal plane inclination in the frontal view has not been studied. Therefore, the aim of this study was to evaluate the aesthetic perception of different degrees of occlusal plane inclination in frontal the view in a mesofacial, brachyfacial and dolichofacial face by orthodontists, dentists and laypeople.

The null hypothesis of our work was: “The aesthetic assessment of different degrees of inclination of the occlusal plane in faces with different biotypes does not differ between orthodontists, general dentists and laypeople”.

## Materials and methods

This study was approved by the Research Ethics Committee of the University of Murcia, Spain. (CEI 4611). All research was performed in accordance with relevant guidelines/regulations and with the Declaration of Helsinki.

### Image selection and manipulation

A frontal face photograph of a smiling woman with mesofacial skeletal pattern was chosen from the digital archives of the Orthodontic Teaching Unit of the University of Murcia. Informed consent was obtained from the patient to manipulate and use her image in this study.

The photo was imported and edited with the software Adobe Photoshop 2020 and Adobe Illustrator 2020 (Adobe Systems Inc, San Jose, Calif). Facial asymmetries and imperfections were corrected, and the image was modified to obtain a face with ideal vertical proportions in the frontal plane; so that the vertical height of the middle third of the face (from the plane to the supraorbital ridges to the base of the nose) was the same as the lower third of the face (from the base of the nose to the lower part of the chin). This image was then modified to obtain a dolichofacial face (vertical height of the lower facial third higher than the middle third) and a brachyfacial face (vertical height of the lower third of the face smaller than the middle third). Finally, for each facial biotype, taking the bipupillary line as reference, images were created with occlusal plane inclinations of 0º, 2º and 4º.

### Catalogue and survey

**Fig. 1:**
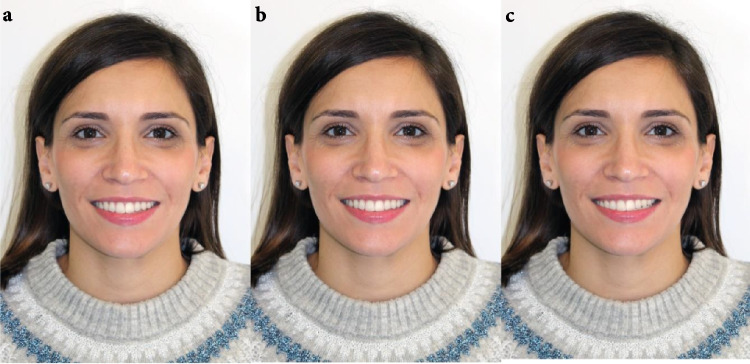
Mesofacial face: **a**) Inclination of 2º in the occlusal plane, **b**) Inclination of 0º in the occlusal plane, **c**) Inclination of 4º in the occlusal plane

**Fig. 2:**
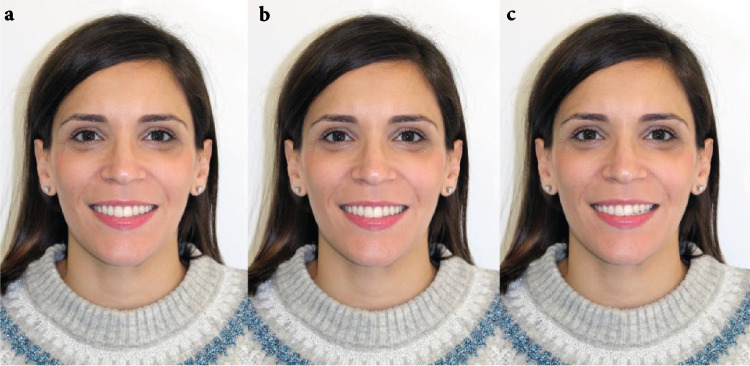
Dolichofacial face: **a**) Inclination of 2º in the occlusal plane, **b**) Inclination of 0º in the occlusal plane, **c**) Inclination of 4º in the occlusal plane

**Fig. 3:**
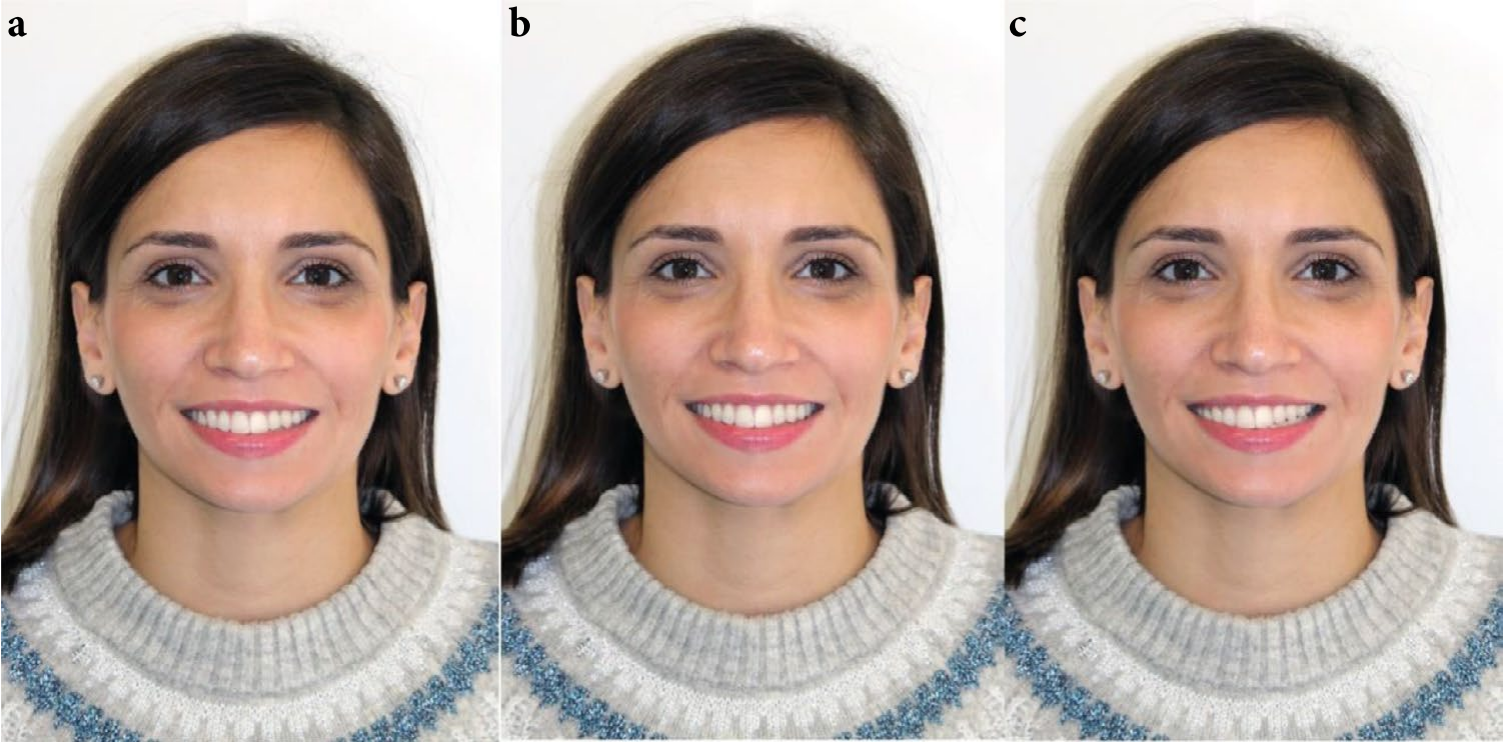
Brachyfacial face: **a**) Inclination of 2º in the occlusal plane, **b**) Inclination of 0º in the occlusal plane, **c**) Inclination of 4º in the occlusal plane

A catalogue was produced with photographic paper (A3 format) containing three pages. The first one showed the three images, with different occlusal plane inclination, of the face with ideal vertical facial proportions (Fig. 1), the second one included those of the dolichofacial face (Fig. 2) and the third one those of the brachyfacial face (Fig. 3). For the three vertical patterns, the first image had an occlusal plane inclination of 2°, the second 0° and the third 4°.

As for the survey, the following data was recorded in a first section: gender, age and profession. The scores for each image were then entered and assigned a score from 1 to 10, “1” being poor aesthetics, and “10” very satisfactory aesthetics.

The presentation of the catalogue and the collection of the survey data was carried out by the same researcher (J.C.S). First, the evaluators were told that they were going to be presented with three sequences of images and how they were to evaluate them aesthetically. They were told that they had 1 min to evaluate each sequence and that they were not allowed to go back to the previous one.

### Evaluators

The photographs were analysed by three groups of evaluators: orthodontists, dentists and laypeople.

We decided to accept an effect size of 1.03, with a value of α ≤ 0.016 and a power of 80% (1 – β ≥ 0.8). Applying these considerations to the G*Power 3.1 program for Mac OS, for the Wilcoxon-Mann–Whitney test (two groups) used in our research, we obtained a sample size corresponding to 25 individuals in each group.

Each group consisted of 25 members (16 women and 9 men) with a mean age of 40 ± 1.2 years old.

### Method error

Ten individuals from each group of assessors re-evaluated the images 21 days after the first assessment. The Wilcoxon test for paired samples revealed no significant differences between the first and second ratings of the images for any of the three groups (*p* > 0.05).

### Statistical analysis

For each facial type, the scores given for the different degrees of occlusal plane inclination by each group of assessors were compared. Comparisons were also made between the scores of the three groups of assessors for the different degrees of occlusal plane inclination. The data were analysed with the programme SPSS 19.0 (IBM SPSS Inc., New York, USA). The Kolmogorov–Smirnov test (*p* < 0.05) and the Levene homogeneity test of variances (*p* < 0.05) were applied, as there was neither homogeneity of variances nor a normal distribution, data were analysed using the Kruskal–Wallis (*p* < 0.05) and the Mann–Whitney test for two independent samples. To avoid an accumulation of errors due to multiple comparisons, the significance level was modified dividing this (*p *< 0.05) between the number of comparisons made (Bonferroni Correction) and *p* < 0.016 was considered significant.

## Results

The results obtained in the meso- and dolichofacial faces were similar (Table 1 and Table 2). In both facial types, orthodontists rated the image with an occlusal plane inclination of 0º as significantly more aesthetic than the images with an inclination of 2º (mesofacial *p* = 0.004, dolichofacial *p* < 0.001) and 4º (mesofacial *p* < 0.001, dolichofacial *p* < 0.001). They also considered the image with an inclination of 2° to be significantly more aesthetic than that of 4º (mesofacial *p* < 0.001, dolichofacial *p* < 0.001). As for dentists and laypeople, it was observed that they rated 0º and 2º inclinations as significantly more aesthetic than 4º inclinations (mesofacial *p* < 0.001, dolichofacial *p* < 0.001). No significant differences were observed between the ratings given to 0º and 2º inclinations (mesofacial: dentists *p* = 0.170, laypeople *p* = 0.040; dolichofacial: dentists *p* = 0.024; laypeople *p* = 0.017). When comparing the ratings of the three groups of evaluators for each inclination of the occlusal plane, it was observed that there were no significant differences between the three groups for the images with 0° (mesofacial *p* = 0.830, dolichofacial *p* = 0.576) and 2º (mesofacial *p* = 0.202, dolichofacial *p* = 0.115). However, the image with 4º was rated by orthodontists as significantly less aesthetic than laypeople (mesofacial *p* = 0.001, dolichofacial *p* = 0.005), while no significant differences were found between orthodontists and dentists (mesofacial *p* = 0.163, dolichofacial *p* = 0.251), nor between dentists and laypeople (mesofacial *p* = 0.042, dolichofacial *p* = 0.063).

**Table 1:** Assessment of the degree of inclination of the occlusal plane in the mesofacial face

	0º			2º			4º		
	Mean ± S.D.	Median		Mean ± S.D.	Median		Mean ± S.D.	Median	
Orthodontists	8.04 ± 1.39	8	a	6.96 ± 1.36	7	b	4.28 ± 1.81	4	Ac
Dentists	8.16 ± 1.02	8	a	7.52 ± 1.50	8	a	5.08 ± 1.75	5	b
Laypeople	8.24 ± 1.33	8	a	7.56 ± 1.15	7	a	6.08 ± 1.55	6	Bb

*S.D*.: Standard deviation Data were analyzed with the Kruskal–Wallis (*p* < 0.05) and the Mann–Whitney test for two independent samples (Bonferroni correction, *p *< 0.016)For each row different lower-case letters indicate the presence of significant differences. For each column different capital letters indicate the presence of significant differences. Unmarked values showed no significant difference with any other

**Table 2:** Assessment of the degree of inclination of the occlusal plane in the dolichofacial face

	0º			2º			4º		
	Mean ± S.D.	Median		Mean ± S.D.	Median		Mean ± S.D.	Median	
Orthodontists	7.76 ± 1.23	8	a	6.24 ± 1.09	7	b	3.92 ± 1.93	4	Ac
Dentists	7.48 ± 1.55	8	a	6.60 ± 1.47	7	a	4.60 ± 1.84	4	b
Laypeople	8.08 ± 1.18	8	a	6.96 ± 1.54	7	a	5.52 ± 1.61	5	Bb

*S.D*.: Standard deviation Data were analyzed with the Kruskal–Wallis (*p *< 0.05) and the Mann–Whitney test for two independent samples (Bonferroni correction, *p* < 0.016)For each row different lower-case letters indicate the presence of significant differences. For each column different capital letters indicate the presence of significant differences. Unmarked values showed no significant difference with any other

Regarding the brachyfacial face (Table 3), orthodontists, dentists and laypeople rated the images with an occlusal plane inclination of 0º and 2º as significantly more aesthetic than those with a 4º inclination (orthodontists *p* < 0.001, dentists *p* < 0.001, laypeople *p* < 0.001) considering similarly the 0º and 2º inclinations (orthodontists *p* = 0.046, dentists *p* = 0.051, laypeople *p* = 0.343). No significant differences were detected when comparing the scores of the three groups for each inclination of the occlusal plane (0º *p* = 0.356, 2º *p *= 0.077 and 4º *p* = 0.163).

**Table 3:** Assessment of the degree of inclination of the occlusal plane in the brachyfacial face

	0º			2º			4º		
	Mean ± S.D.	Median		Mean ± S.D.	Median		Mean ± S.D.	Median	
Orthodontists	6.60 ± 1.32	7	a	5.76 ± 1.45	6	a	3.48 ± 2.00	4	b
Dentists	6.88 ± 1.66	7	a	5.92 ± 1.52	6	a	4.00 ± 1.60	4	b
Laypeople	7.36 ± 1.93	7	a	6.76 ± 1.48	6	a	4.64 ± 1.86	4	b

*S.D.*: Standard deviation Data were analyzed with the Kruskal–Wallis (*p* < 0.05) and the Mann–Whitney test for two independent samples (Bonferroni correction, *p *< 0.016)For each row different lower-case letters indicate the presence of significant differences. For each column different capital letters indicate the presence of significant differences. Unmarked values showed no significant difference with any other

## Discussion

The aim of this study was to analyse the aesthetic perception by orthodontists, dentists and laypeople of different occlusal plane inclinations in the frontal view in relation to facial pattern.

We used frontal images of a full smiling face rather than isolated images of a smile or the lower third of the face, because this perspective of the face is the most usual during any social interaction [1].

When studying the influence of facial pattern on the aesthetic perception of smiles, some authors have used faces of different people with different vertical patterns in their assessments [9]. However, other studies [5, 7, 8], like us, digitally modified the face of the same individual and obtained images of all three vertical patterns. In this way our aim was to avoid the influence of other facial features such as eyes, nose, etc., on aesthetic perception.

Our results showed that for dentists and laypeople, facial pattern had no impact on the aesthetic perception of the occlusal plane as they rated it equally for all three facial types, considering 0° and 2° occlusal plane inclinations similar and significantly more aesthetic than 4º.

The inclination of the occlusal plane in the frontal view is related to the horizontal axis of the face, it may be that in our study both the dentists and the laypeople consciously or unconsciously took this as reference in the evaluation of the images with the three facial types, thus diluting the impact of facial pattern. Chang et al. found that for laypeople, the sex or attractiveness of the model did not influence the assessment of occlusal plane inclination. The characteristics of the face neither diminished nor enhanced the aesthetic perception of this variable. However, both sex and attractiveness did affect smile features unrelated to the horizontal axis of the face, such as the degree of gingival smile, overbite, or the size of the buccal corridors [1].

Orthodontists agreed in their assessments of occlusal plane inclination in meso- and dolichofacial faces, but not in the brachyfacial face. For meso- and dolichofacial pattern faces, they were more selective than dentists and laypeople when assessing this variable, considering the occlusal plane inclination of 0º as being significantly more aesthetic than that of 2º and 4º; in addition, the image with an inclination of 2º was evaluated as significantly more aesthetic than that of 4º. In the brachyfacial face, they were more tolerant of the inclination of the occlusal plane than in the other two vertical patterns, since they valued 0° and 2° inclinations in a similar way and considered 4° inclination to be significantly less aesthetic. In other words, for orthodontists, the mesofacial and dolichofacial pattern did not influence the evaluation of the occlusal plane and they were more demanding in their determinations than dentists and laypeople, probably due to their greater specialisation; however, in the brachyfacial face, the orthodontist's level of tolerance increased, and the aesthetic evaluations were similar to those of the dentists and laypeople.

We cannot compare our results with other studies since, to our knowledge, the influence of facial pattern on the perception of the occlusal plane has not been evaluated. However, Huang et al. observed in orthodontists a higher level of tolerance with bigger buccal corridors in a brachyfacial face than in a dolichofacial face [11]. Therefore, it seems that the brachyfacial pattern does influence in the orthodontist’s assessment of a smile, this vertical pattern attenuating the alteration of some of its characteristics.

We observed amongst orthodontists, dentists and laypeople that the mean scores given to the brachyfacial face were lower than those given to the other two biotypes. There is no consensus in the literature as to which biotype is considered more aesthetic, probably because the evaluations also depend on the attractiveness of the model. Macías Gago, Romero Maroto and Crego observed that in men considered attractive, the facial pattern was more horizontal than in women [19]. Williams, Rinchuse and Zullo found that for a male face, the brachy- and dolichofacial patterns were found to be less aesthetically pleasing than the mesofacial pattern. However, for a female face, both brachyfacial and dolichofacial patterns were found to be more pleasing than the mesofacial pattern [8].

Regardless of the facial pattern, our results showed that the occlusal plane inclination of 4° was perceived by all three groups as the most unaesthetic, being assessed in the meso- and dolichofacial pattern by the orthodontists as significantly less aesthetic than by laypeople. Dentists were in an intermediate position, as their evaluations did not differ from those of orthodontists or laypeople. Olivares et al., in a study with isolated images of smiles with inclinations of 0º, 2º and 4º in the occlusal plane also obtained that the image of 4º was considered to be the most unaesthetic by orthodontists, dentists and laypeople. However, orthodontists rated it as significantly more aesthetically negative than dentists and laypeople (with no significant differences between dentists and laypeople) [15]. It may be that by introducing the full face in this work, the horizontal axis reference helped the dentists to detect plane inclination more easily in the frontal view and therefore in this study their scores more closely resemble those of the orthodontists. Padwa et al., who assessed occlusal plane inclination in a full face, mentioned that 4º was the limit for recognising the presence of canting by 90% of the observers, both trained observers and untrained observers [16]. Other studies, also of full faces, observed that laypeople were not able to distinguish occlusal plane inclinations of less than 3º [17, 20]. While Ker et al., using isolated photographs of smiles observed that laypeople accepted an occlusal plane inclination of up to 4º as aesthetic, although a third of the respondents accepted an occlusal plane inclination of up to 6º [14].

Considering our results, the null hypothesis of our work was rejected, because the aesthetic assessment of different degrees of inclination of the occlusal plane in faces with different biotypes differ between orthodontists, general dentists and laypeople.

The degree of maxillary canting plays a crucial role in treatment decisions, with orthodontic treatment generally recommended for mild cases, while severe cases may require orthognathic surgery. Our results showed that the facial pattern influences the perception of the occlusal plane. Understanding these differences in aesthetic perception across various population groups is essential for clinicians, as it can enhance diagnostic accuracy and optimize treatment planning, ultimately leading to more personalized and effective orthodontic care.

Among the limitations of our study is that we only used a female face, and the aesthetic assessment of the facial pattern may be different from that of a male face [8, 19], so in further studies it would be interesting to include a male face in the assessments. On the other hand, we should consider that the face evaluated was that of a Caucasian individual. It would be interesting to assess faces from other racial backgrounds as well.

## Conclusions

- The facial pattern had no impact on the aesthetic perception of the occlusal plane for the dentists and laypeople, as they assessed it in the same way in the three facial types, considering 0º and 2º occlusal plane inclinations similar and significantly more aesthetic than 4º.
-Orthodontists agreed in their assessments of occlusal plane inclination in the meso- and dolichofacial faces, where they were more demanding than dentists and laypeople, considering 0º occlusal plane inclination as significantly more aesthetic than 2º and 4º. The 2° inclination was rated as significantly more aesthetic than the 4° inclination. However, in the brachyfacial face, they were more tolerant than in the other two vertical patterns, their evaluations being similar to those of dentists and laypeople.
- The inclination of the occlusal plane of 4° was perceived by the three groups of evaluators as the most unaesthetic, being in the meso- and dolichofacial patterns considered to be significantly less aesthetic by orthodontists than by laypeople.

## Data Availability

No datasets were generated or analysed during the current study.
